# Biological Effects of Monoenergetic Carbon Ions and Their Associated Secondary Particles

**DOI:** 10.3389/fonc.2022.788293

**Published:** 2022-02-17

**Authors:** Dylan J. Buglewicz, Kade D. Walsh, Hirokazu Hirakawa, Hisashi Kitamura, Akira Fujimori, Takamitsu A. Kato

**Affiliations:** ^1^ Department of Environmental & Radiological Health Sciences, Colorado State University, Fort Collins, CO, United States; ^2^ Department of Charged Particle Therapy Research, National Institutes for Quantum and Radiological Science and Technology, Chiba, Japan; ^3^ National Institute of Radiological Sciences, National Institutes for Quantum and Radiological Science and Technology, Chiba, Japan

**Keywords:** DNA damage, carbon ion radiotherapy, Bragg peak, gamma-H2AX, secondary particles

## Abstract

DNA double-strand breaks (DSBs) are the main factor behind carbon-ion radiation therapy (CIRT)-induced cell death. Nuclear interactions along the beam path between the primary carbon ions and targets result in nuclear fragmentation of carbon ions and recoiled particles. These secondary particles travel further distances past the Bragg peak to the tail region, leading to unwanted biological effects that may result in cytotoxicity in critical organs and secondary induced tumors following CIRT. Here, we confirmed that the density of the DSB distributions increases as the cell survival decreases at the Bragg peak and demonstrated that by visualizing DSBs, the various LET fragmentation ions and recoiled particles produced differences in their biological effects in the post-Bragg peak tail regions. This suggests that the density of the DSBs within the high-LET track structures, rather than only their presence, is important for inducing cell death. These results are essential for CIRT treatment planning to limit the amount of healthy cell damage and reducing both the late effect and the secondary tumor-associated risk.

## Introduction

The most consequential of the ionizing radiation-induced DNA damage lesions are DNA double-strand breaks (DSBs). These DSBs are known to be the major factor responsible for radiation-induced cell death when left unrepaired or misrepaired (1, 2). However, misrepair of DSBs may give rise to genomic instability, thus increasing the risk of cancer development (3). High linear energy transfer (LET) radiation includes alpha particles, carbon, and iron ions which deposit their energy within densely ionizing tracks that are created by the particle’s traversal through the cell. This allows for the formation of multiple close-proximity DNA damages including DSBs, single-strand breaks (SSBs), and base damages following high LET irradiation. Such complex DNA damage has also been demonstrated in prior studies using clusters of γ-H2AX foci as a surrogate marker for DSBs by horizontal irradiation and high-resolution microscopy (4–6). Additionally, these clustered DSBs are known to be very difficult for the cell to repair and may be a strong contributor to genomic instability (7, 8).

Carbon ion radiotherapy (CIRT) has been effective for cancer treatment due to its excellent dose distribution with maximized dosage at the Bragg peak (9). As the carbon ions approach their Bragg peak, their LET values increase, and the DNA damage qualities become more complex. Carbon ions interact with matter along their beam path, and this results in fragments and recoiled particles, such as hydrogen, helium, lithium, beryllium, and boron, that also provide doses on the beam path (10, 11). Specifically at the Bragg peak, these secondary particles consist of 50% of the doses (12). However, those secondary particles can travel longer ranges than the primary carbon ions and provide significant doses past the Bragg peak, thus producing a tail region (12). CIRT provides more dose at the tail region than seen with proton radiotherapy, as proton fragments that have lower energy are thus limited in range as compared to CIRT. In previous studies, we presented the cytotoxic effect in the post-Bragg peak tail region of carbon ions in CHO and its DNA repair mutant cells as well as in human cancer cells (13–15). Other recent reports, using a horizontal irradiation and analysis system, also visually demonstrated the cytotoxic effects following proton and carbon-ion irradiation (15, 16). DNA damage responses in micrometer order scale sensitivity have also revealed high LET like foci track damage following proton irradiation (17). It is important to address the cytotoxicity in the post-Bragg peak tail region as it may cause unwanted side effects in CIRT through cellular loss or accumulation of mutations. Currently, evidence of secondary tumor production following CIRT has not been demonstrated (18). However, if the post-Bragg peak region contains DNA damage produced by high LET radiation, it may cause long-term effects in CIRT patients. Thus, it is important to address the biological effects within the carbon-ion post-Bragg peak tail region as the current radiobiology information in this region is limited.

To address these issues and appropriately observe the biological effects from carbon-ion irradiation, we have developed a method capable of observing the DSB distribution within the full monoenergetic carbon-ion beam range including the post-Bragg peak tail region in a single biological system using γ-H2AX foci as a marker for DSBs. One of the major advantages of our method of irradiation, in which the beam source is parallel to the cell culture flasks, is that it makes it possible to view the DNA damage-induced foci along high-LET particle tracks, which would not be readily observed if the incident particles were perpendicular to the cell culture flask base to which the cells are attached. To the best of the authors’ knowledge, this is the first study in which the DSB distribution for the full carbon-ion beam range including the tail region has been evaluated in a single *in vitro* biological system. Here we not only demonstrate that our system is capable of identifying the depths within the beam range that show characteristics of high-LET radiation but also demonstrate how the DSB distribution changes as the beam approaches the Bragg peak and can observe the heterogeneity of the DNA damage in the post-Bragg peak depths resulting from the secondary particles.

## Materials and Methods

### Cell Culture and Irradiation Conditions

Chinese hamster ovary (CHO) cells were kindly supplied by Dr. Joel Bedford (Colorado State University, Fort Collins, CO). Cells were grown and maintained in α-MEM (Invitrogen, Carlsbad, CA, USA) supplemented with 10% heat-inactivated fetal bovine serum (Sigma, St Louis, MO, USA), supplemented with antibiotics and antimycotics at 37°C in incubators at 5% CO_2_ and 100% humidity. Doubling times were approximately 12 h for this cell line. Carbon ions and iron ions were accelerated to 290 and 500 MeV/nucleon, respectively, using the Heavy Ion Medical Accelerator in Chiba (HIMAC) synchrotron at the National Institute of Radiological Sciences (NIRS), Chiba, Japan. Dose rates for carbon ions and iron ions were set at 1 Gy/min. The irradiation field is within 2.5% uniformity (19). Monoenergetic 290-MeV/nucleon carbon ions and 500-MeV/nucleon iron ions have LET values of 13 and 200 keV/μm on entrance, respectively. Cell culture flasks or chamber slides were set up in a horizontal position (approximately 5°) to carbon-ion or iron-ion beam source, respectively, prior to irradiation. X-ray irradiation was performed at 200 kVp and 20 mA with aluminum (0.5 mm)–copper (0.5 mm) filters (Shimadzu, TITAN-320, NIRS), and dose rates were set at 0.5 Gy/min. Irradiations were carried out at room temperature. The beam characteristics and dosimetry using HIMAC have been described previously (20, 21). In brief, dosimetry of the carbon ions was obtained using a combination of an ionization chamber and a fluence measurement by a gas flow-type multiwire proportional counter. The dose-averaged LET values were calculated by HIBRAC code.

### Irradiation Procedure for Cell Survival Assays

**Figure 1:**
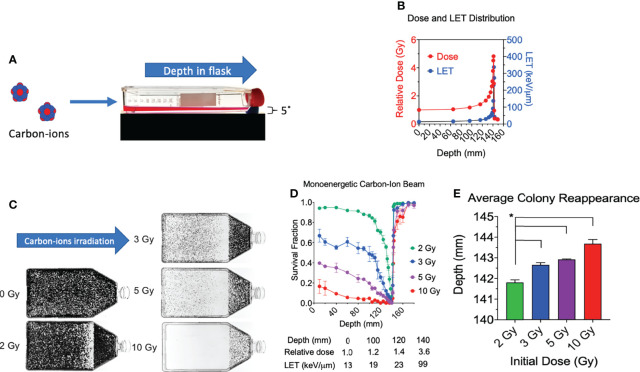
Cell survival vs. depth of the full monoenergetic carbon-ion beam range at increasing initial doses in CHO cells. **(A)** An illustration of the horizontal irradiation setup. **(B)** Dose and LET distribution of carbon-ion 290 MeV/n. **(C)** Images of T-175 cell culture flasks following irradiation of 2, 3, 5, or 10 Gy initial dosage. **(D)** Survival fraction vs. depth following irradiation of each initial dosage. Statistical significances are illustrated in **Supplemental figure 1**. **(E)** Average colony reappearance depth at each initial dosage for the detailed analysis of cytotoxicity after Bragg peak. * indicates p < 0.05. Error bars indicate standard errors of the means from two or three independent experiments per each initial dosage.

Cultured cells were trypsinized and resuspended into growth medium. 60 ml of media containing 30,000 cells was placed into a T-175 cell culture flask a few hours prior to irradiation, and attachment was confirmed. Cells were irradiated at room temperature with the dose rate of 1 Gy per minute. All flasks were irradiated independently with an incident dosage of either 2, 3, 5, or 10 Gy directly at beam entry. The flasks rested flat on the cell culture area, and the beam entry point was at the bottom of the flask (non-capped end) (**Figure 1A**) (15). Immediately following irradiation, all cells were incubated for a period of 7 days for colony formation. After this culturing period, each culture flask was then washed with 0.9% NaCl, fixed in 100% ethanol, and stained with 0.1% crystal violet.

### Survival Fraction Calculation for Cell Survival Assays

Survival fractions were calculated as described previously (15). In short, to quantify the survival fraction at each of our evaluated depths, they were scored for every millimeter along the width of the flask either possessing a surviving colony, defined as a colony containing >50 cells, or not possessing a surviving colony and the average value was calculated. Therefore, the survival fraction was calculated as the number of colonies at the specific depth divided by total millimeter along the width of the flask. This approach was repeated for a minimum of three independent experiments for each one of our initial dosages of 2 and 3 Gy with the reference at the 0-Gy dose, and two independent experiments for 5 and 10 Gy. The post-Bragg peak narrow cell culture area near the cap did not interfere with the survival fraction, as previously reported (15, 17).

To evaluate the cytotoxicity of the post-Bragg peak area in detail, a colony reappearance analysis was carried out as described previously (15, 17). The reappearance of colony formation following the Bragg peak was recorded with a ruler. Colony reappearance was defined as the average distance from the entrance for the first observable colonies after the Bragg peak. Seven equally spaced locations were analyzed for each flask to obtain a sensitive analysis of the extension of the cytotoxic range after the Bragg peak.

### Irradiation Procedure for γ-H2AX Assays

For carbon-ion and X-ray irradiations: The top of each T-225 cell culture flask, i.e., the portion of the flask opposite to the cellular adherent side, was removed by a hot knife, and 6 poly-L-lysine-coated glass microscope slides were placed inside each flask in a sterilized manner and oriented, as indicated in **Supplemental Figure 2**. The positions of the slides were chosen to maximize the width and distance of the observable space within the flask. Tops were then returned to their respective flask and sealed with parafilm prior to addition of CHO cells. Cultured cells were trypsinized and resuspended into the growth medium. 60 ml of media containing 9 million cells was placed into T-225 cell culture flasks a few hours prior to irradiation, and attachment was confirmed. For iron-ion irradiations, 0.4 ml of media containing 50,000 cells was placed into each well of an 8-well Chamber Slide System (Thermo Fisher, Waltham, MA, USA) a few hours prior to irradiation and attachment was confirmed. Chamber slides were placed with 4 of the 8 wells (one side) directly facing the iron-ion beam in a horizontal orientation (approximately 5 degrees) relative to the iron-ion beam. Only wells of the side directly facing the iron-ion beam were analyzed to appropriately represent the incident iron ions (22). Carbon ions were irradiated and analyzed by this method to address the variations within the full carbon-ion beam range capable within our system while iron ions utilized the method to address only their ions upon the entrance region to maintain a high LET positive control with their LET value being 200 keV/μm.

### Immunofluorescence Staining

A DSB marker, γ-H2AX foci formation assay was carried out as it was previously (23). Briefly, each slide containing cells was taken out of the flask following irradiation and was washed in cold PBS and fixed for 15 min in 4% w/v paraformaldehyde in PBS and washed again in PBS. Cells were then permeabilized for 5 min in 0.2% v/v Triton X-100 (Sigma, St Louis MO, USA) in PBS and washed twice in PBS. Slides were treated with 10% goat serum for 1 h at 37°C for blocking. Antibodies were diluted with 10% v/v goat serum in PBS. Cells were incubated with 1:300 diluted mouse anti-γ-H2AX antibody (Millipore, Burlington, MA, USA) for 1 h at 37°C, washed three times in PBS, and incubated with 1:500 diluted Alexa Fluor 488 goat anti-mouse IgG antibody (Abcam, Cambridge, MA, USA) for 1 h at 37°C, and washed four times in PBS. DAPI (4′,6-diamidino-2-phenylindole) (Roche, Indianapolis, IN, USA) in SlowFade was then applied to stain the DNA.

### γ-H2AX Foci Analysis by Zeiss Axioplan Microscopy With and Without MetaMorph Deconvolution

Microscopic images were captured with a Zeiss Axioplan microscope using a ×60 objective. 20 slices of images with every 0.5 μm were obtained to cover 2–3 μm of CHO thickness to quantify γ-H2AX foci within the 3-dimensional nucleus in a 2-dimensional image. 20 deconvoluted or non-deconvoluted images were stacked into a single-layer image to analyze γ-H2AX foci. Non-deconvoluted stacked images were analyzed for foci intensities. Deconvoluted stacked images were analyzed for foci cluster sizes and the number of individual foci. For this analysis, images taken by the Zeiss Axioplan were deconvoluted and processed using MetaMorph software (Molecular Devices, LLC, San Jose, CA, USA) *via* 2D no-neighbor deconvolution.

### γ-H2AX Foci Scoring

Foci scoring was carried out blindly in each experiment with at least 50 cells/each depth analyzed or at least 50 cells/each control. Unless stated otherwise, all foci analyses are reported followed by the mean and standard error of the means from at least 3 independent experiments. Foci cluster sizes within the cells at each depth or in controls were scored as >2.4 µm in length and/or width. Foci length was measured within MetaMorph software following image processing. Foci of this size were chosen due to the largest observed foci size in our negative control, unirradiated cells, which were determined to be 0.8 μm, with a very low frequency of occurrence, and very few clusters within the cells following our high-dose low-LET control irradiation, 4-Gy X-ray, were observed as >2.4 μm. In contrast, following our high-LET control, 2-Gy iron ion, many clusters were observed as >2.4 μm. Additionally, a prior study using a higher image resolution than that used in our study demonstrated the presence of many individual γ-H2AX foci within each cluster following 2-Gy iron-ion beam irradiation (4). Thus, with use of our conventional image resolution *via* a Zeiss Axioplan microscope with deconvolution, we analyzed the individual foci number per cell (**Supplemental Figure 3**). However, the manual foci counting was difficult to reproduce due to highly clustered foci. To eliminate possible counting bias, the intensity values of each cell within each depth was obtained from the stacked image without deconvolution. Apparent, clear-tracked foci were scored as a track. Because depths lower than 90 mm possessed marginal track figures, the data were not included. In addition, depths were also defined by the relative depth percentage with respect to the physical dose peak at 141.4 mm which we defined as the relative depth = 100%.

### Statistical Analysis

All experimental data were derived from at least 3 independent experiments with exception to 5- and 10-Gy cell survival, whose data were derived from 2 independent experiments. For experiments involving γ-H2AX foci, at least 50 cells per each depth or control per experiment were analyzed. Statistical significance was determined by using one-way analysis of variance (ANOVA) followed by the Bonferroni multiple-comparison test by GraphPad Prism 8 software (GraphPad, La Jolla, CA, USA). p < 0.05 was considered as statistical differences for all tests. Analysis was carried out between entry depth (10 mm) versus others unless stated otherwise, as with post-Bragg peak depths compared to the final depth (155 mm) versus others.

## Results

### Physics Nature of Monoenergetic Carbon Ions 290 MeV/n

All experimental flasks were irradiated in a horizontal orientation with a carbon-ion beam source with 290 MeV/n (**Figure 1A**), as it was conducted in our previous research (15). The monoenergetic carbon ions have an initial LET value of 13.4 keV/µm and reaches 337 keV/µm at the depth of 142 mm (**Figure 1B**). The Bragg peak is at 141.4 mm. When initial 2 Gy of monoenergetic carbon ions is irradiated, the dose at the Bragg peak is approximately 9.6 Gy. The post-Bragg peak tail region at 148.8 mm is estimated to have 0.7 Gy.

### Cytotoxicity of the Full Range of Monoenergetic Carbon Ions 290 MeV/n

Consistent with our prior work, we observed a decrease in survival at our first evaluated depth with an increase in initial beam entry irradiation dosage (15). Under initial beam irradiation doses of 2, 3, 5, and 10 Gy at the entrance within the flask, the distance of 140 mm presented a clear cytotoxicity without colonies (**Figure 1C**) and the lowest relative survival score (**Figure 1D**). The absorbed dose at this narrow area is more than 4 times that at the entrance dose. A clear cytotoxicity near the Bragg peak, 50% reduction of relative survival fraction compared to the initial depth of 10 mm, was observed at 132 mm for 2 Gy, 115 mm for 3 Gy, 100 mm for 5 Gy, and 40 mm for 10 Gy. The post-Bragg peak cytotoxicity, defined as survival score less than 0.9, was observed up to 142 mm for 2 Gy, 143 mm for 3 Gy, 145 mm for 5 Gy, and 160 mm for 10 Gy (**Figure 1D**).

To clarify the potential cytotoxicity at the post-Bragg peak, a detailed analysis of cytotoxicity after the Bragg peak was carried out by the distance for the reappearance of colonies. Our results also demonstrated that the average depth of colony reappearance following no colonies at the Bragg peak at 140 mm extended as the initial irradiation dosage increased (**Figure 1E**). Reappearance of colony formation was observed for the initial dosages of 2, 3, 5, and 10 Gy at the depths of 141.80 ± 0.22, 142.64 ± 0.19, 142.91 ± 0.04, and 143.69 ± 0.35 mm, respectively. The depths of colony reappearance following the Bragg peak at 140 mm for 3, 5, and 10 Gy were all found to be significant as compared to the initial dosage of 2 Gy (p < 0.05). These findings demonstrate that the depth of maximum cytotoxicity is consistent throughout a wide range of initial dosages and suggest that secondary particles of carbon ions may be responsible for cell death at the post-Bragg peak region as these fragmented ions and recoiled products are known to be capable of traveling greater distances with relatively the same velocity (24).

### γ-H2AX Foci Distribution in the Full Range of Monoenergetic Carbon Ions 290 MeV/n

**Figure 2:**
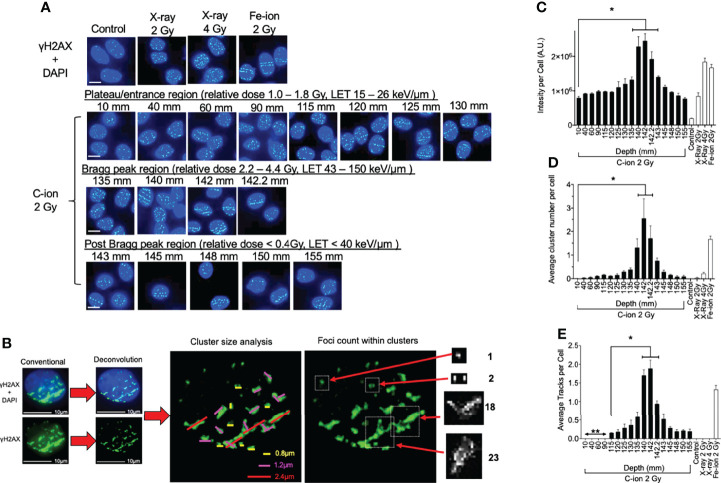
γ-H2AX foci formation at each depth following 0.5 h post monoenergetic 290 MeV/n carbon-ion irradiation in CHO cells. **(A)** Representative images of carbon-ion-induced foci at the different depth, control, X-ray, and iron-ion irradiation. Green indicates γ-H2AX foci. Blue is DAPI-stained nuclei. Indicator bar represents 10 µm. **(B)** Deconvolution process with MetaMorph software prior to analysis and method representation of foci cluster size scoring and foci counting in each cell. Foci clusters were scored as greater than 0.8, 1.2, or 2.4 µm in width and/or length represented as yellow, pink, or red lines, respectively. Foci counting was determined by differences in pixel intensity across each cluster. **(C)** Intensity analysis of foci at different depths. **(D)** Clustered (diameter > 2.4 µm) foci analysis at the different depth. **(E)** Track foci analysis at the different depth. Bonferroni multiple-comparison test, * indicates p < 0.05. Error bars indicate the standard errors of the means from a minimum of 50 cells analyzed per each depth per experiment and at least three independent experiments per each irradiation treatment.

To further investigate the biological effects on a molecular level behind our results obtained in the clonogenic assays, we observed the DSB distribution *via* the γ-H2AX assay at increasing depths within the carbon-ion beam range including the post-Bragg peak tail region. For all γ-H2AX assay experiments, we utilized an exponentially growing CHO10B2 cell line as with our survival experiments. Cells were analyzed for γ-H2AX expression at 0.5 h following their respective irradiation, as prior studies have indicated that the strongest induction of γ-H2AX foci was observed at this time point (4, 25, 26). For experimental controls, we used unirradiated cells as the negative control, 2 and 4 Gy X-ray irradiation for low-LET positive controls, and 2 Gy iron-ion irradiation for the high-LET positive control (**Figure 2A**). We utilized 2-Gy carbon-ion irradiation to address the DSB distribution within the full beam range capable in our system (10–155 mm), as this initial beam irradiation dosage demonstrated the largest survival fraction ratio between its survival fraction at the entrance (10 mm) to the Bragg peak depth (140 mm) in our clonogenic assays. For each individual experiment, at least 50 cells were analyzed at each depth as well as in each of our controls. As with our clonogenic assay, the statistical significance of each depth in these experiments and for controls was determined by comparison to the first evaluated depth closest to the beam entry at 10 mm.

Representative images show that quantities and qualities of γ-H2AX foci changed on the path of carbon-ion irradiation (**Figure 2A**). Foci analysis was carried out using three different categories including signal intensity per cell without deconvolution and the number of clusters of foci per cell, and foci track number per cell after image processing with deconvolution **(** **Figure 2B**). The signal intensity was a less subjective analysis than manual counting of foci. An initial 2 Gy of carbon-ion induced signal intensity of 7.9 × 10^5^ AU (arbitrary unit) at a 10-mm entrance from a background of 1.9 × 10^5^ AU. It was maximized at 142 mm (relative depth = 100.4%) with 24.5 × 10^5^ AU. At the post-Bragg peak region, signal intensity decreased to 7.6 × 10^5^ AU at 155 mm (relative depth = 110.7%); however, it was statistically higher than the background and had a similar value to the signal intensity at the entrance **(** **Figure 2C**).

For the qualitative analysis of foci, we used foci cluster size and track analysis. Foci cluster size was initially analyzed to determine high LET-specific cluster size. Cluster size was measured using MetaMorph software following appropriate image calibration. A cluster size smaller than 0.8 µm was observed in all tested samples including unirradiated or low LET X-ray-irradiated controls. Cluster sizes of more than 1.2 µm were not observed for the unirradiated control but were in all irradiated samples including low LET X-ray irradiation (**Supplemental Figure 4**). However, the cluster size of more than 2.4 µm was considered as a high LET signature in this analysis as it was highly observed within our high LET control, iron ion, as well as in the carbon-ion Bragg peak region, with very few being observed within our low LET control, X-ray. There were more than 0.25 clusters per cells between 130 and 145 mm (relative depths = 91.9 and 102.5%, respectively) following carbon-ion irradiation (**Figure 2D**). At 142 mm, the number of clusters was peaked with 2.5 clusters per cell. These large clusters were also observed at the post-Bragg peak region up to 155 mm with reduced numbers, but still greater than those observed at the entrance depth of 10 mm.

Linear foci tracks were visually noticeable near the Bragg peak or high LET iron-ion irradiation with the horizontal irradiation method (**Figure 2A**). Track structures were observed following 2 Gy of carbon-ion irradiation between 115 and 155 mm and high LET iron ion with strong confidence, but not in the cells of the control, low LET X-ray irradiation, or carbon ion at the entrance region. A distance of up to 90 mm and shorter presented non-confident foci tracks, which may be a false positive of randomly distributed foci. Therefore, the actual track count was conducted beginning from 115 mm (relative depth = 81.3%). The average number of tracks peaked at 142 mm with 1.9 tracks per cell (**Figure 2E**). The average number of tracks above 0.5 was observed from 135 to 145 mm. These quantitative and qualitative analyses of foci distribution strongly suggested that DNA damage is peaked at 142 mm. This value is similar to the maximum cytotoxicity observed at 140 mm and matched well with the physical dose and LET distribution of the Bragg peak.

### Differentiating the High-LET Track Structures Between Track-Positive Cells of Post-Bragg Peak Depths

**Figure 3:**
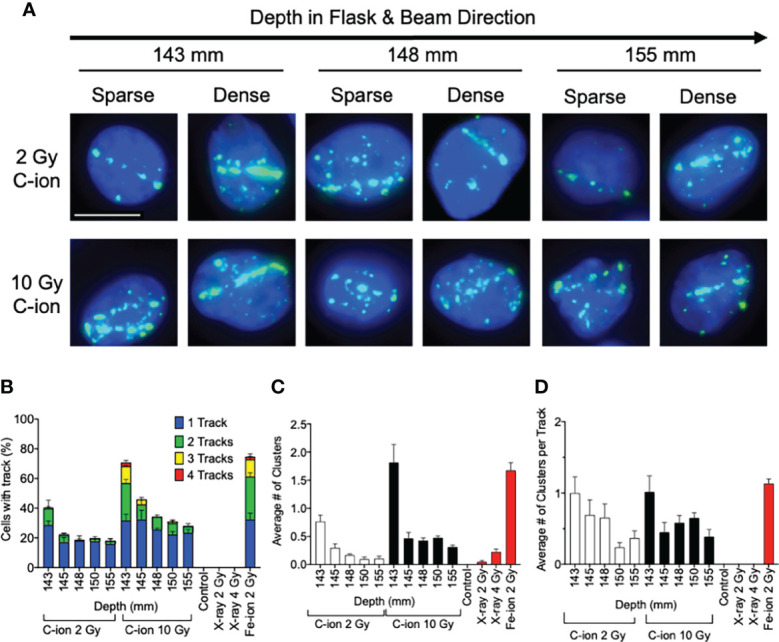
Analysis of post-Bragg peak depths following 2- and 10-Gy carbon-ion irradiation. **(A)** Representative images of foci track structure variation (sparse or dense) at the post-Bragg peak following either 2 or 10 Gy. **(B)** Percent of cells with 0, 1, 2, 3, or 4 foci tracks in the post-Bragg peak. **(C)** Average number of clustered (diameter > 2.4 µm) foci in the post-Bragg peak. **(D)** Average number of clustered foci in the track in the post-Bragg peak. Error bars indicate standard errors of the means from a minimum of 50 cells analyzed per depth per experiment and at least three independent experiments per irradiation treatment.

At the post-Bragg peak region following 2 Gy of carbon-ion irradiation, the foci analysis showed a steep decrease, but we observed a significantly higher signal intensity and high LET signature compared to the unirradiated control (**Figures 2C–E**). To further investigate the biological effects after the Bragg peak tail region, an in-depth analysis was conducted by adding 10 Gy of carbon-ion irradiation. While high-LET foci track structures were detected in all post-Bragg peak depths (143–155 mm, relative depth = 102.1%–110.7%) following either 2- or 10-Gy carbon-ion irradiation (**Figure 3A**), track and cluster numbers decreased by a further post-Bragg peak **(** **Figures 3B, C** **)**.

Additionally, visible differences of the track structures were distinctively observed after the Bragg peak up to 155 mm as sparse and dense tracks. To characterize these foci tracks, tracks containing a greater number of multiple cluster foci defined as >2.4 μm cluster foci size were analyzed. Such tracks with cluster foci were greatly observed at 143 mm, but they were dramatically decreased after 145 mm (**Figure 3C**). The number of large clusters in tracks between 2 and 10 Gy did not show statistically significant changes (**Supplemental Figure 5**). This means that the high LET-induced damage fraction is higher near the Bragg peak and lower after 145 mm, up to 155 mm in this study, which is dominated by lower LET-induced damage. This was likely due to the property differences between each of the carbon-ion nuclear fragmentation ions and recoiled particles, as it has been previously reported that heavy ions produce denser foci distributions within in their tracks than the light ions (27).

### DSB Distribution Correlation With Cell Death

**Figure 4:**
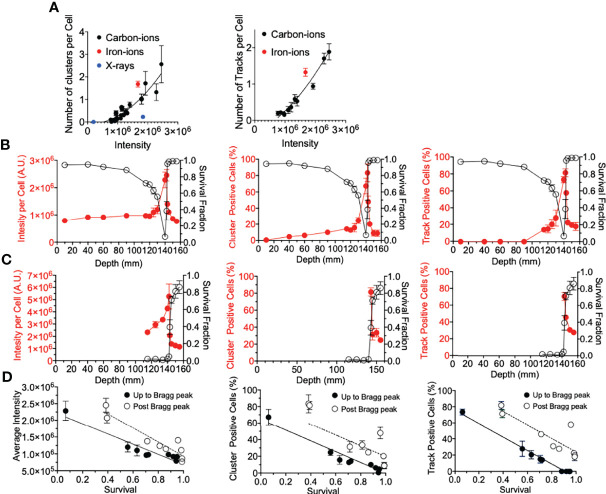
γ-H2AX foci comparison with survival. **(A)** Number of clusters or tracks per intensity per cell. Black, red, and blue circles indicate carbon ions, iron ions, and X-ray irradiation, respectively. **(B)** Gy carbon-ion irradiation showing full beam range comparison of survival with average intensity, percent of cells with clusters, or tracks. **(C)** 10-Gy carbon-ion irradiation showing full beam range comparison of survival with average intensity, percent of cells with clusters, or tracks. **(D)** Correlation between survival and intensity, clusters, and tracks. Up to Bragg peak 10–140 mm (●) and post Bragg peak 142–155 mm (○). Linear regression analysis was carried out with GraphPad Prism 8 software. Error bars indicate standard errors of the means from a minimum of 50 individual cells analyzed per depth per experiment from at least three independent experiments per irradiation treatment.

Firstly, for correlation against DNA damage complexity, clustered foci and tracked foci were analyzed as a function of signal intensity. Both clusters and tracks increased with signal intensity in a quadratic manner (**Figure 4A**). X-ray-induced clusters presented a much lower efficiency to produce clusters per signal intensity. On the other hand, iron ions demonstrated a much higher efficiency to produce clusters or tracks per signal intensity. The efficiency to produce a greater number of clusters or tracks was observed to be associated with the LET values of the radiation. To address the importance of the γ-H2AX foci distribution for the cellular lethality at the post-Bragg peak, cell survival scores were plotted against γ-H2AX signal intensity, cluster foci, and track-positive cells following either 2 or 10 Gy of carbon-ion irradiation (**Figures 4B, C** **)**. Each tested parameter matched well with the survival fraction, although this was not a one-to-one ratio (**Figure 4D**). This was especially observed when entrance and post-Bragg peak data were analyzed separately. The signal intensity showed that the entrance region had more efficiency to kill cells per signal intensity compared to the post-Bragg peak region. Clusters and tracks in the post-Bragg peak were less effective to inactivate cells, but the inactivation ratio was similar between pre- and post-Bragg peak regions. Therefore, all parameters matched well with the survival fraction and initial DNA damage quantities and qualities are highly associated with cell survival.

## Discussion

The biological effects in cytotoxicity and DNA damage of full-range hadron beam were previously conducted utilizing a proton beam with small flasks using a relatively low proton beam energy (17). The current study utilizing the carbon-ion beam required using a much larger flask to cover the longer penetration of carbon ions. The irradiation system was developed from the previously reported cytotoxicity and genotoxicity analysis with the modifications of using multiple slides placed within each experimental flask in order to observe the DNA damage distribution in the full-range carbon-ion beam (15). Our cell survival results were consistent with prior studies describing the characteristic nature of carbon ions having a sharp increase in the deposited dosage the closer the beam approaches to the Bragg peak (13, 15). We observed a sharp decrease in survival fractions the closer the depth was to the Bragg peak, with the lowest survival fraction being observed at 140 mm in depth, regardless of initial treatment dosage, and a slight difference of 2 mm with physical dose distribution (**Figures 1B, C** **)**. Following the Bragg peak, a sharp increase in survival was observed, which suggested that the majority of the dosage was deposited at the Bragg peak. However, there was a notable decrease in survival in the post-Bragg peak regions as the initial irradiation dosage increased (**Figure 1E**). Cellular lethality was also extended after the Bragg peak in a dose-dependent manner, as was previously observed (**Figure 1E**) (15). Several potential reasons may explain this extended cytotoxicity after the Bragg peak. One possibility is due to the heterogeneity of beam quality, e.g., the monoenergetic beam is not perfectly monoenergetic meaning that some fraction of carbon ions may be of higher energy allowing for them to travel further (12). Another possible reason explaining this observation may be due to the secondary particles from nuclear fragmentation of the initial carbon ions and recoiled particles, as these fragmentation ions and recoiled particles are known to be capable of traveling longer distances past the Bragg peak (28). The studies presented that these fragment ions and recoiled particles provide non-negligible doses after the Bragg peak (10, 28). Importantly, prior studies have identified that these secondary particles are composed of proton, helium, lithium, beryllium, and boron. At the beam entrance, carbon particles dominate the relative fluence of the beam particles but the fragmentation reactions lead to an increase in the secondary particles as the beam proceeds toward the Bragg peak. At the Bragg peak, only around 50% of the primary carbon ions are believed to remain. Following the Bragg peak, the number of secondary proton and helium particles exceeds those of the carbon particles and the other secondary particles are observed to be less than 5% of the secondary proton and helium particles (10, 11). To clarify whether the extended toxicity following the Bragg peak is due to the secondary particles rather than beam uniformity, DNA damage distribution was quantitatively and qualitatively analyzed.

The main challenge of this study was the analysis of DNA damage distribution in the full-range carbon-ion irradiation up to 155 mm. To make this possible, flame-sterilized and poly-L-lysine-coated glass microscope slides were tightly placed within large T-175 flasks for cell culture. A DSB marker, γ-H2AX foci formation, was used to determine DNA damage quantities and qualities based on the number and distribution in the cellular nuclei. The number of γ-H2AX foci, which is the quantitative analysis of DNA damage, showed a sharp increase of DNA damage near the Bragg peak and peaked at 142 mm (**Figure 2C**). This may be associated with the fact that high-LET irradiation, such as carbon ions near the Bragg peak, not only produce DSBs but also are efficient at producing complex DSBs in addition to causing cluster of DSBs over larger scales because of correlation of events along the track. Moreover, as the LET of the particles increases, there is an increase in the frequency and complexity of complex DSBs produced. The complexity of DNA damage was analyzed with two parameters of foci distribution including the cluster of multiple foci at one location and foci track structure, both of which are signatures of high LET radiation exposure (29, 30). The kinetics of foci disappearance after high LET radiation exposure is known to be slower than that after low LET radiation. The complexity of DNA damage was also peaked at 142 mm (**Figures 2D, E** **)**. Therefore, 2-mm differences between DNA damage distribution and cellular lethality were observed in both quantitative and qualitative analyses.

This difference could be explained by several reasons. The first is the geometrical difference between flask of survival and flask with cells on slides, as the slides may move during transportation and irradiation within flasks. However, slides were fixed on their location by melting the plastic with heat to avoid potential movement. The second reason can be due to the overkill effect from focused DNA damage to hit cells. LET values increased at the Bragg peak between 140 and 142.5 mm as 91 to 337 keV/mm with a peak at 142 mm with 337 keV/mm (**Figure 1B**). Ionizing radiation most efficiently produces DNA damage and kills cells at around 100 keV/mm. The LET above this value will result in overkill or, in other words, when a single particle deposits much more energy than is required to kill a cell, and this results in it killing less cells per absorbed dose. Moreover, at very high LET values, the percentage of non-hit cells has been observed to increase (31). In contrast to what was observed, we expected the cluster number per cell within the range of the carbon ion to be significantly higher than what was observed at these depths if all of our observed tracks contributed, as this would change the distribution of foci and tracks across the irradiated cell population according to the equation, $D=0.16\times L\times \frac{N}{A}$ , where D is the dose in Gy, L is LET in keV/μm, N is the number of tracks per nucleus, and A is the nuclear area in square micrometers perpendicular to the beam (32).

As observed in survival, cytotoxicity was maximized at 140 mm, where the LET values are near 100 keV/µm and DNA damage was maximized at 142 mm with above 300 keV/µm. Our previous study also showed that the RBE value of cell survival was maximized at 200 keV/μm of iron ions with the value of around 4 and decreased after 250 keV/μm of silicon ions (33).

The last possibility is explained by the time of our analysis. DNA damage analysis was carried out 0.5 h following irradiation. γ-H2AX foci can be maximized at this time point (34, 35), but the lethality is strongly associated with repair capacity and residual DNA damages (17). Typically, higher initial damages cause higher residual damages. Clustered foci after Bragg peak may remain longer than simple foci at the entrance region. Data from 24 h following irradiation may answer this in the future studies as this may be due to a combination of decreased repairability of complex DSB at individual sites of DNA damage, in conjunction with the overlap of multiple γ-H2AX foci associated with the correlation of breaks along the radiation track, requiring multiple sites of damage to be repaired for the clustered foci to be lost. It would also be beneficial in future studies to conduct the chromosomal aberration assay within our system. Comparison between the types of chromosomal aberrations at each depth with our present data may further answer this discrepancy as the proximity of the DSBs along the high-LET tracks may increase the frequency of chromosomal aberrations arising from misrepair between these correlated breaks as well as in the complexity of chromosomal rearrangements arising with non-symmetrical types typically resulting in clonogenic cell death (36, 37).

The analysis of DNA damage at the post-Bragg peak provided validation of our system. The cluster and track foci were observed at the post-Bragg peak region. If the extended cell death or DNA damage at the post-Bragg peak region came from heterogenic energy of initial carbon ions or possible irradiation errors, the complex type of DNA damage should be observed at any depth analyzed randomly. However, both clustered foci or track foci structures were observed up to 155 mm following 2 or 10 Gy of carbon-ion irradiation (**Figures 3B, C** **)**. When comparing 2 and 10 Gy data, we expected the number of cells with tracks and the average number of clustered foci to increase by a factor of 5 which was not the observed case and may indicate that we did not observe all the tracks, meaning it may require more than the 150 cells per depth analyzed to provide more accurate results in this case. However, evaluating the quality of the track, not the dose, in which we normalized the average number of clusters by track allowed us to overcome this problem by evaluating per track not per cell. Thus, we observed that the cluster positive foci tracks, the clear signature of high LET radiation exposure, were gradually decreased after Bragg peak (**Figure 3D**). This suggests that DNA damage within the post-Bragg peak region is mainly from the secondary particles including nuclear fragmentations and recoiled particles rather than random carbon-ion beam artifact. Since the complexity of DNA damage was observed in the post-Bragg peak region, the lighter fragments travel further and potentially a few centimeters after the Bragg peak and some of the secondary particles have LET values more than 10 keV/μm (11, 12). This study conducted up to 155 mm based on the limitation of slide placement in the T175 flask size, but the secondary particles may travel at more than 10% of the initial Bragg peak range of 140 mm. Thus, the secondary particles from carbon ions were observed to travel much longer than the secondary particles observed after proton irradiation in our previous study (17). Furthermore, simulation analysis also supports these findings (28, 38). Our study clearly suggests that the post-Bragg peak of carbon ions contains a small but significant amount of high LET radiation fraction, which is enough to cause the cytotoxicity and potentially genotoxicity (**Figures 1E**, **2A**).

Lastly, we determine if the underlying factor between the foci distribution correlation with cell death was due to the amount of foci per cell (*via* average intensity per cell), the foci distribution per cell (*via* percentage of cluster >2.4 µm positive cells), the presence of a track structure (*via* percentage of track positive cells), or a combination of these (**Figure 4**). All three factors explained well for cell survival. While there was not a one-to-one correlation between each of these factors and cell survival, the importance of this analysis was that the relationship between γ-H2AX and survival was very similar between pre-Bragg peak and post-Bragg peak regions (**Figure 4**). This suggests that the DNA damage and cellular death after irradiation at the post-Bragg peak occur by the same mechanisms, which depends on the DSB quantities and qualities, as at the pre-Bragg peak regions.

In conclusion, the DSB distribution analysis of the full carbon-ion beam range in a single biological system conducted in this study clarified differences in the DNA damage distribution near the Bragg peak, as the γ-H2AX distribution is dramatically changed in their number and character near the Bragg peak. The cellular lethality was confirmed in the post-Bragg peak region, and this can be explained by the DSBs produced by the various LET nuclear fragments and recoiled particles as observed in the foci track structures. The signature of DNA damage from secondary particles was observed in the tail region far from the Bragg peak at least up to a 10% distance of the initial Bragg peak. These results are of great interest as the DSBs at the post-Bragg peak region may contribute to cellular death and organ dysfunction and even genetic instability, possibly resulting in cancer cell propagation. Results of this study should be carefully considered during radiation treatment planning to limit the amount of healthy cell damage in patients. As our system of irradiation also demonstrated that the biological response can dramatically change in millimeter differences, a limitation to the current irradiation system is that the biological response is critically dependent on both the dose delivered and radiation quality (LET), both of which also vary significantly with depth and therefore position on the flask. Thus, to further address the variation in relative biological effectiveness in the post-Bragg peak region and relate this to the spectrum of particles and associated energies of the fragments, more detailed cell survival studies with cells plated at a range of depth with the beam normal to the plated cell population should also be performed at and beyond the Bragg peak, backed up with detailed modeling of the carbon ion and associated fragments as a function of the depth in future studies.

## Data Availability Statement

The original contributions presented in the study are included in the article/**Supplementary Material**. Further inquiries can be directed to the corresponding author.

## Author Contributions

Conceptualization, TK. Methodology and formal analysis, DB, KW, HK, and TK. Resource data curation, DB, KW, HH, HK, AF, and TK. Writing—original draft preparation, DB and TK. Writing—review and editing, DB, KW, HK, AF, TK. Funding acquisition, AF, TK. All authors contributed to the manuscript revision and read and approved the submitted version.

## Funding

This research was partially funded by Dr. Akiko Ueno Radiobiology Fund (TK) and the Japan Ministry of Education, Culture, Science and Technology (MEXT) Grants-in-Aid for Scientific Research on Innovative Areas (JP15K21745, AF).

## Conflict of Interest

The authors declare that the research was conducted in the absence of any commercial or financial relationships that could be construed as a potential conflict of interest.

## Publisher’s Note

All claims expressed in this article are solely those of the authors and do not necessarily represent those of their affiliated organizations, or those of the publisher, the editors and the reviewers. Any product that may be evaluated in this article, or claim that may be made by its manufacturer, is not guaranteed or endorsed by the publisher.

## Supplementary Material

Supplementary Figure 1 Survival fraction vs depth following irradiation of each initial dosage. Red and blue arrows indicate survival fraction at 142 and 143 mm, respectively, to demonstrate how survival fraction decreases in the post Bragg peak at increased initial irradiation treatment dosage. Areas highlighted in gray or green represent a significant decrease (P<0.05) or significant increase (P<0.05) compared to the closest analyzed depth near beam entry at 1.0 cm, Bonferroni multiple comparisons test.

Supplementary Figure 2 Depiction of slide placement to determine beam depth in flask following carbon-ion irradiation.

Supplementary Figure 3 Foci number per cell after initial 2 Gy of carbon-ion irradiation. Counting was conducted manually with deconvoluted images. Red dots indicate data from individual cells. Mean and standard deviation are shown.

Supplementary Figure 4 Number of clusters per cell after initial 2 Gy of carbon-ion irradiation. Cluster sizes were divided into up to 0.8 µm, between 0.8-1.2 µm, and larger than 2.4 µm. Depiction of slide placement to determine beam depth in flask following carbon-ion irradiation.

Supplementary Figure 5 Cluster size within tracks after initial 2 Gy or 10 Gy of carbon-ion and 2 Gy of iron-ion irradiation. Cluster sizes were divided into up to 0.8 µm, between 0.8-1.2 µm, and larger than 2.4 µm. Depiction of slide placement to determine beam depth in flask following carbon-ion irradiation.
