# Supplementary figures and images for: Biological Effects of Monoenergetic Carbon Ions and Their Associated Secondary Particles
Source: Front Oncol. 2022 Feb 17;12:788293. doi: 10.3389/fonc.2022.788293 (PMC8892238; doi:10.3389/fonc.2022.788293)

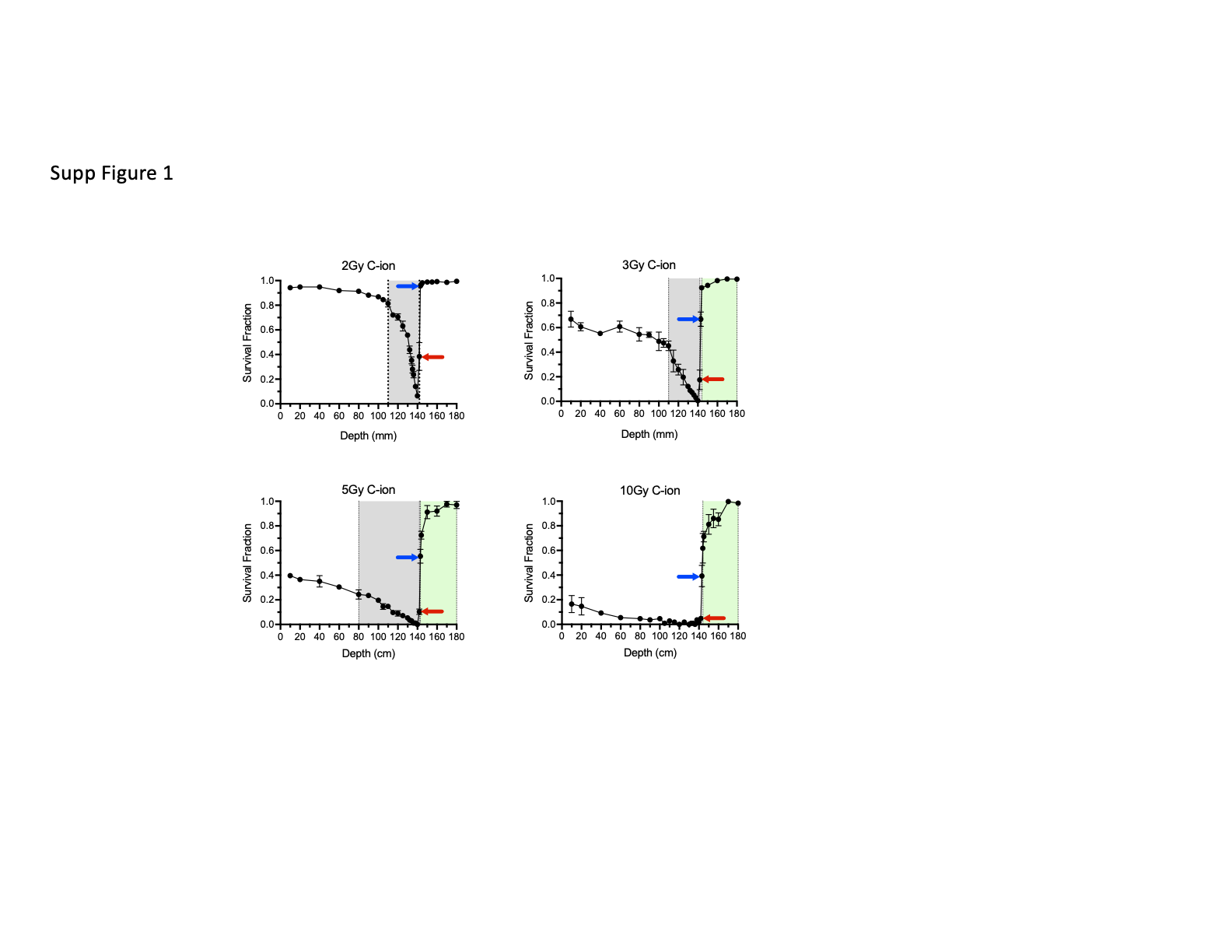

Supplement: Supplementary Figure 1 — Survival fraction vs depth following irradiation of each initial dosage. Red and blue arrows indicate survival fraction at 142 and 143 mm, respectively, to demonstrate how survival fraction decreases in the post Bragg peak at increased initial irradiation treatment dosage. Areas highlighted in gray or green represent a significant decrease (P<0.05) or significant increase (P<0.05) compared to the closest analyzed depth near beam entry at 1.0 cm, Bonferroni multiple comparisons test. [file Image_1.tiff]

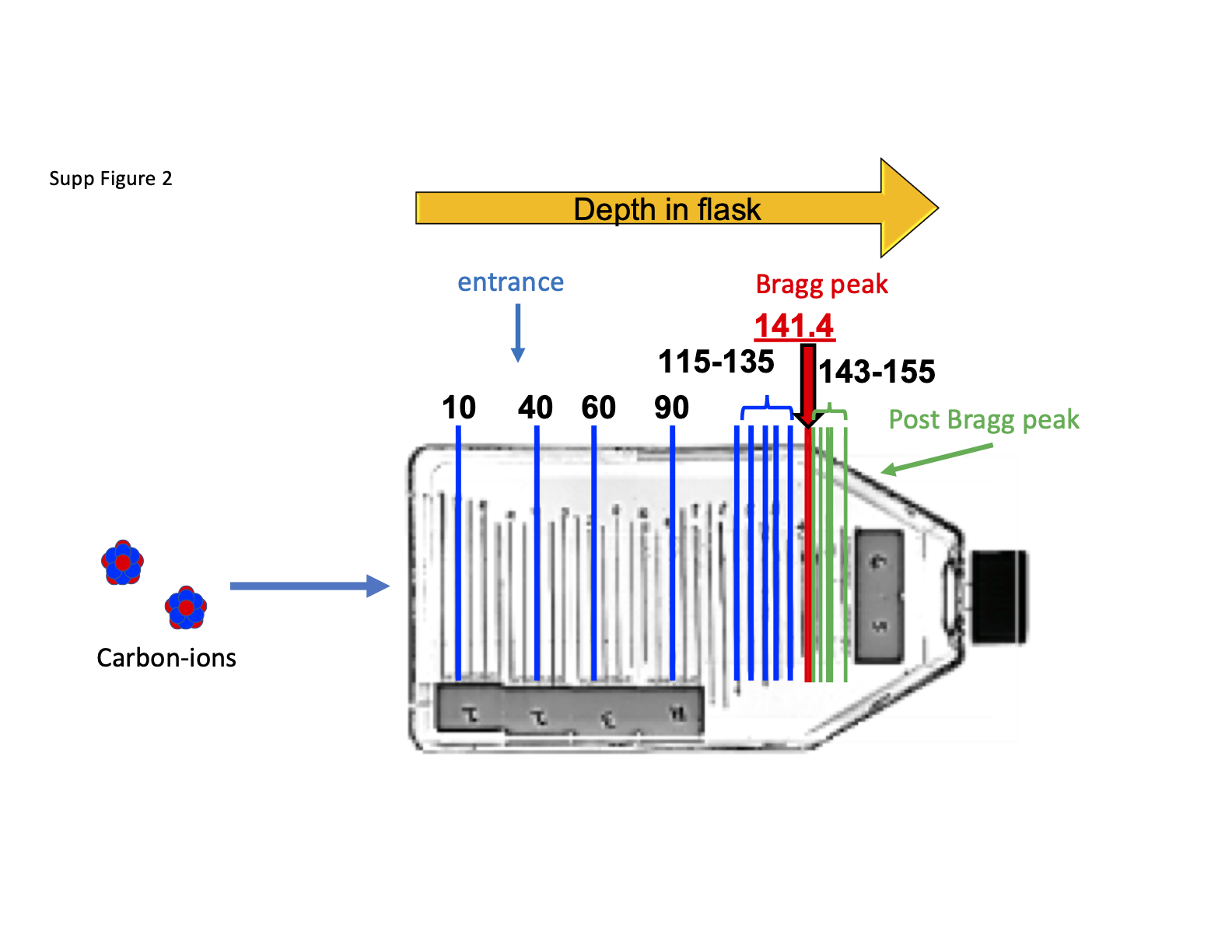

Supplement: Supplementary Figure 2 — Depiction of slide placement to determine beam depth in flask following carbon-ion irradiation. [file Image_2.tiff]

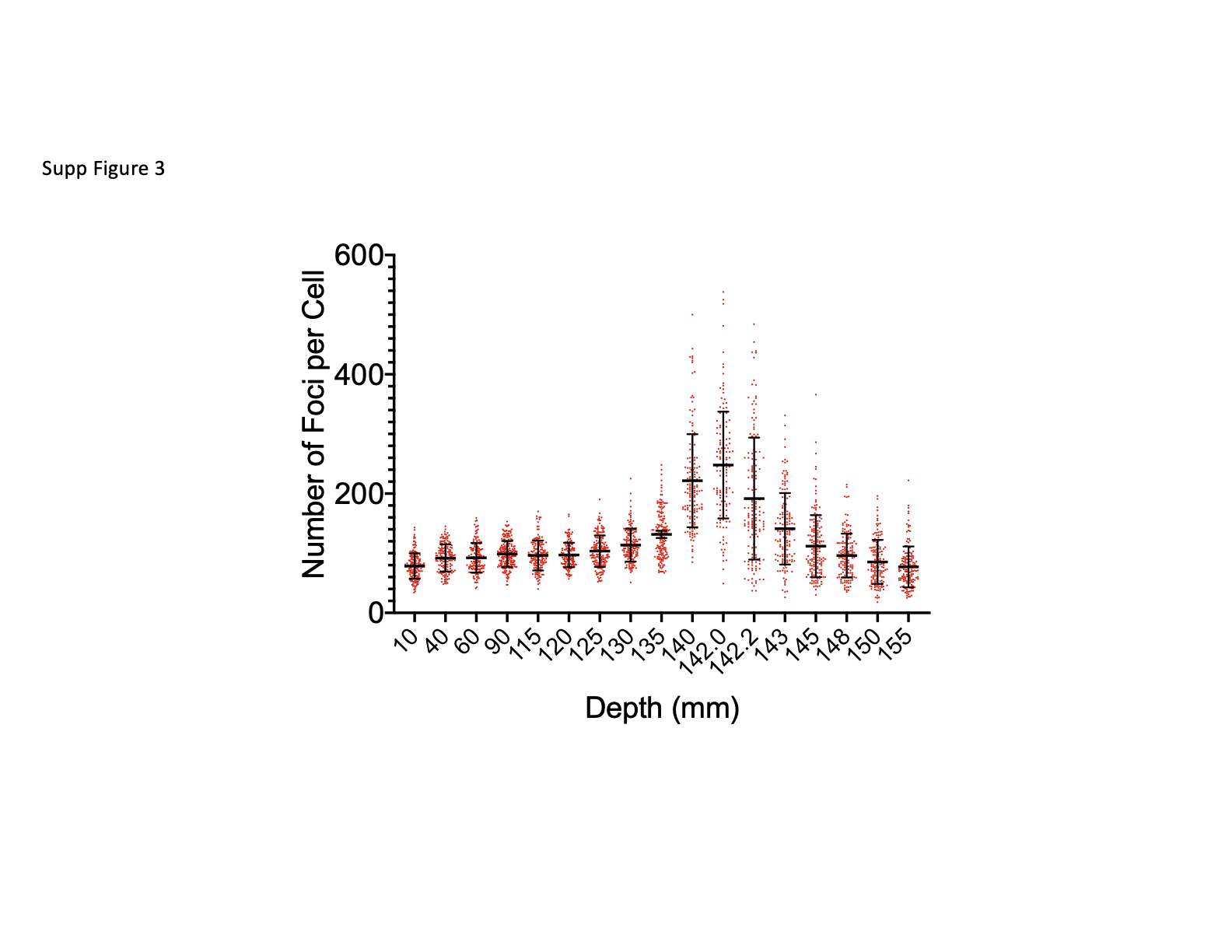

Supplement: Supplementary Figure 3 — Foci number per cell after initial 2 Gy of carbon-ion irradiation. Counting was conducted manually with deconvoluted images. Red dots indicate data from individual cells. Mean and standard deviation are shown. [file Image_3.tiff]

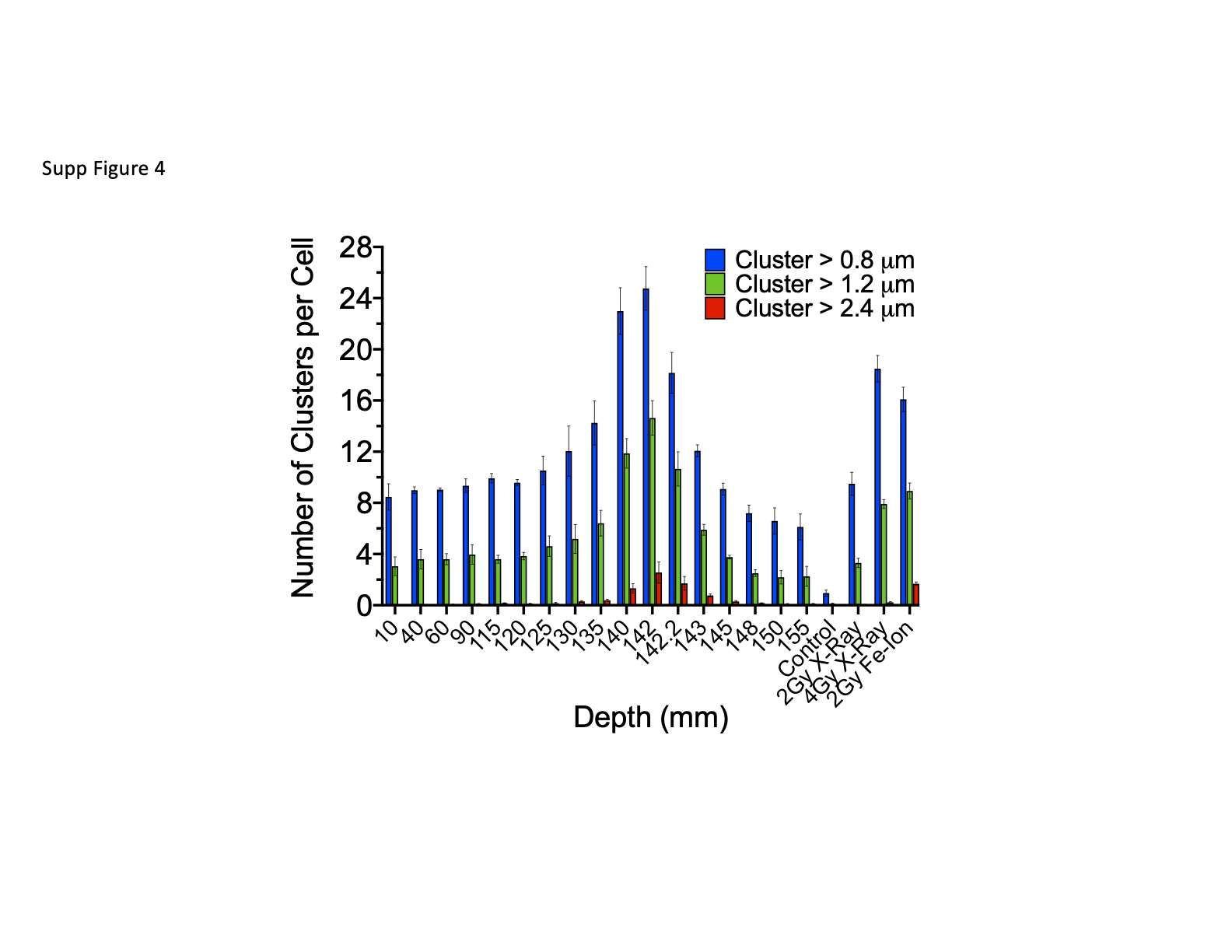

Supplement: Supplementary Figure 4 — Number of clusters per cell after initial 2 Gy of carbon-ion irradiation. Cluster sizes were divided into up to 0.8 µm, between 0.8-1.2 µm, and larger than 2.4 µm. Depiction of slide placement to determine beam depth in flask following carbon-ion irradiation. [file Image_4.tiff]

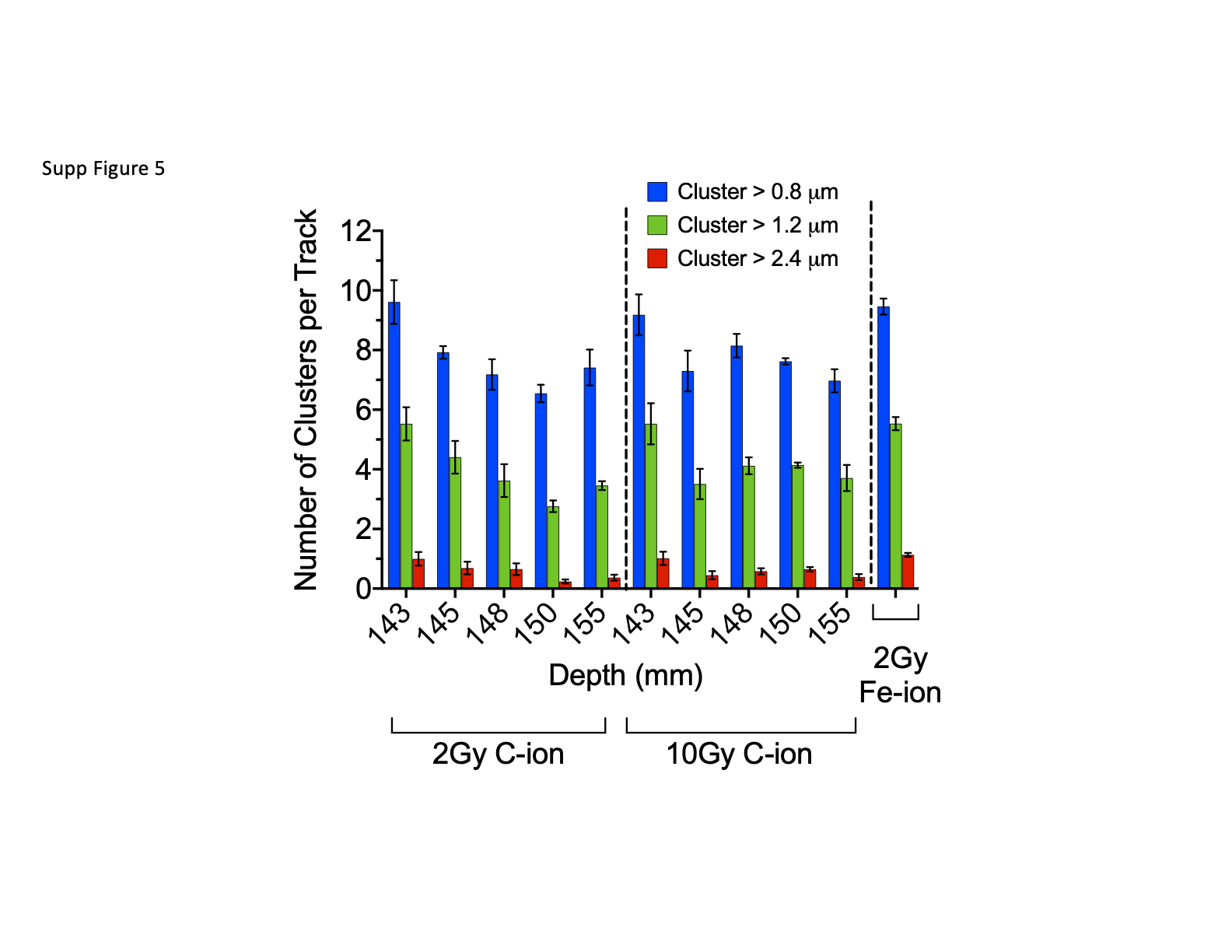

Supplement: Supplementary Figure 5 — Cluster size within tracks after initial 2 Gy or 10 Gy of carbon-ion and 2 Gy of iron-ion irradiation. Cluster sizes were divided into up to 0.8 µm, between 0.8-1.2 µm, and larger than 2.4 µm. Depiction of slide placement to determine beam depth in flask following carbon-ion irradiation. [file Image_5.tiff]
